# L1CAM overexpression promotes tumor progression through recruitment of regulatory T cells in esophageal carcinoma

**DOI:** 10.20892/j.issn.2095-3941.2020.0182

**Published:** 2021-06-15

**Authors:** Xuan Zhao, Shasha Liu, Xinfeng Chen, Jianyi Zhao, Feng Li, Qitai Zhao, Tan Xie, Lan Huang, Zhen Zhang, Yu Qi, Yang Yang, Song Zhao, Yi Zhang

**Affiliations:** 1 Biotherapy Center, The First Affiliated Hospital of Zhengzhou University, Zhengzhou 450052, China; 2 Cancer Center, The First Affiliated Hospital of Zhengzhou University, Zhengzhou 450052, China; 3 Department of Thoracic Surgery, The First Affiliated Hospital of Zhengzhou University, Zhengzhou 450052, China; 4 Department of Pediatric Surgery, The First Affiliated Hospital of Zhengzhou University, Zhengzhou 450052, China; 5 School of Life Sciences, Zhengzhou University, Zhengzhou 450052, China; 6 Henan Key Laboratory for Tumor Immunology and Biotherapy, Zhengzhou 450052, China

**Keywords:** L1CAM, CCL22, Tregs, TGF-β, esophageal squamous cell carcinoma

## Abstract

**Objective:**

L1 cell adhesion molecule (L1CAM) exhibits oncogenic activity in tumors. However, the link between L1CAM and the tumor microenvironment remains poorly understood in patients with esophageal squamous cell carcinoma (ESCC). In this study, we investigated how L1CAM expression in ESCC affects the oncogenic characteristics of tumor cells and the tumor microenvironment.

**Methods:**

Human ESCC samples were collected, and the mRNA and protein levels of L1CAM were examined by real-time PCR and immunohistochemistry. Overexpression and knockdown gene expression assays were used for mechanistic studies. The cell proliferation and cell cycle were measured with CCK-8 assays and flow cytometry. Cell migration and invasion ability were measured with Transwell assays. Multiplex bead-based assays were performed to identity the factors downstream of L1CAM. Xenograft studies were performed in nude mice to evaluate the effects of L1CAM on tumor growth and regulatory T cell (Treg) recruitment.

**Results:**

L1CAM expression was significantly elevated in ESCC tissues (*P* < 0.001) and correlated with poorer prognosis (*P* < 0.05). Ablation of L1CAM in ESCC cells inhibited tumor growth and migration, and increased tumor cell apoptosis (*P* < 0.05). In the tumor microenvironment, L1CAM expression correlated with Treg infiltration in ESCC by affecting CCL22 secretion. Mechanistically, L1CAM facilitated CCL22 expression by activating the PI3K/Akt/NF-κB signaling pathway. Furthermore, CCL22 promoted Treg recruitment to the tumor site; the Tregs then secreted TGF-β, which in turn promoted L1CAM expression *via* Smad2/3 in a positive feedback loop.

**Conclusions:**

Our findings provide new insight into the mechanism of immune evasion mediated by L1CAM, suggesting that targeting L1CAM-CCL22-TGF-β crosstalk between tumor cells and Tregs may offer a unique means to improve treatment of patients with ESCC.

## Introduction

Esophageal cancer (EC) is the third most commonly diagnosed cancer, and it has the fourth highest mortality rate^1^ in China. Esophageal squamous cell carcinoma (ESCC) is the main pathological type of EC, accounting for 95% of EC cases^2^. The 5-year overall survival (OS) ranges from 15% to 25%, and the best outcomes are associated with early diagnosis^3^. Patients diagnosed at earlier stages have substantially better outcomes than those diagnosed at later stages. Surgery, radiotherapy, and chemotherapy are the main options, but their curative effects have not been satisfactory. Therefore, new therapeutic strategies and molecular targets are urgently needed^4^.

The tumor immunosuppressive microenvironment, an important factor in tumor immune escape, is composed of various immunosuppressive cells, the tumor vasculature, and tumor-associated-stromal cells. Cytokines and chemokines have been shown to play crucial roles in this process^5,6^. Tumor-intrinsic signaling pathways have important roles in oncogenesis, and emerging evidence suggests that these pathways are involved in regulating the tumor microenvironment (TME) and tumor immune escape^7^. Our previous studies have demonstrated that lung adenocarcinoma-intrinsic glycogen branching enzyme (GBE1) signaling regulates T cell infiltration and programmed cell death ligand 1 (PD-L1) expression in tumor cells; therefore, GBE1 may be a promising target for achieving tumor regression through cancer immunotherapy^8^. We have also reported that a novel oncogenic gene *MEALSTROM* accelerates tumor progression through recruiting myeloid derived suppressor cells by upregulating interleukin (IL-8) expression in ESCC^6^.

L1 cell adhesion molecule (L1CAM), a member of the L1 family of adhesion molecules, belongs to the immunoglobulin superfamily^9^. L1CAM was originally identified as a key molecule in the development of the nervous system. Several recent studies have revealed that the expression of L1CAM plays a pivotal role in the course of cancer development. For example, strong L1CAM expression has been found in ovarian carcinoma and uterine adenocarcinoma, and identified as a marker predictive of short survival^10^. Preoperative evaluation of L1CAM levels in curettage or plasma samples has been found to predict lymph node metastasis and patient prognosis in endometrial cancer^11^. *L1CAM* has also been identified as a target gene of Wnt-β-catenin signaling in colorectal cancer and detected at the invasive front of cancer tissue^12^.

*L1CAM* is a newly and important cancer-associated gene in several malignant tumors. However, the potential oncogenic function and molecular mechanisms of L1CAM in ESCC are unknown. The primary objectives of the present study were to assess the expression and function of L1CAM in ESCC. More importantly, we aimed to validate the underlying molecular mechanisms through which L1CAM regulates the TME of ESCC.

## Materials and methods

### Patients and tumor samples

All fresh ESCC tissue samples were obtained from the Thoracic Surgery Department of the First Affiliated Hospital of Zhengzhou University, China. Formalin-fixed, paraffin-embedded tissues were obtained from the pathology department of the same hospital. All samples were pathologically confirmed, and none of the patients had undergone radiation, chemotherapy, or any other therapy before being enrolled in this study. A total of 106 fresh ESCC tissues (typically 1 cm^3^) with paired adjacent normal tissues (located more than 5 cm away from the tumor tissues) were washed with PBS and cut into small pieces. Some of these were used to examine *L1CAM* mRNA expression, and some were processed for flow cytometry analyses (with a BD FACSCanto II instrument). Sixty-nine formalin-fixed, paraffin-embedded tissues were used to examine L1CAM protein expression. All patients provided written informed consent before tissues or associated clinical information were collected, and this research was approved by the Institutional Ethical Committee of the First Affiliated Hospital of Zhengzhou University (2018-KY-92).

### Cell lines and cell cultures

Human ESCC cell lines (EC1, KYSE70, TE1, and KYSE450), an immortalized esophageal epithelial cell line (Het-1α), and human embryonic kidney epithelial 293T cells (293T) were obtained from the Cellbank Shanghai Institutes for Biological Sciences of the Chinese Academy of Sciences. All cells were cultured in DMEM (Hyclone, Logan, UT, USA) supplemented with 10% fetal bovine serum (Hyclone, Logan, UT, USA), 100 U/mL penicillin, and 100 μg/mL streptomycin at 37 °C and 5% CO_2_ in a humidified incubator.

### RNA extraction and quantitative real-time polymerase chain reaction

Total RNA was extracted from ESCC cells or tissues with TRIzol reagent (Thermo Fisher Scientific Inc., Waltham, MA, USA) according to the manufacturer’s instructions. RNA purity and concentration were quantified with a NanoDrop 2000 spectrophotometer (Thermo Fisher Scientific Inc.), and 1 μg of total RNA was used to synthesize the first-strand cDNA with a PrimeScript RT reagent Kit with gDNA Eraser (TaKaRa Bio, Beijing, China). qRT-PCR was performed to detect the mRNA with SYBR Premix Ex Taq II (TaKaRa Bio, Beijing, China) on an Mx3005P System (Agilent, Santa Clara, CA, USA). Glyceraldehyde-3-phosphate dehydrogenase (GAPDH) was used as an endogenous control. The 2^-ΔΔCt^ method was used to calculate the expression levels.

### Immunohistochemistry (IHC)

IHC was used to detect protein expression, and immunohistochemical staining was performed as previously described^13^. Anti-L1CAM antibody (Santa Cruz Biotechnology, USA), anti-CCL22, and anti-FOXP3 antibodies (both Abcam, Cambridge, Cambridgeshire, United Kingdom) were used at dilutions of 1:100, 1:200, and 1:200, respectively. After incubation with horseradish peroxidase-conjugated secondary antibody for 1 h at 37 °C, sections were treated with substrate 3,3-Diaminobenzidine (DAB) and counterstained with hematoxylin and visualized under a microscope (Leica, USA). Protein expression was scored according to the percentage of positively stained tumor cells and the intensity of staining. The percentage of stained tumor cells was scored as follows: 0 (no positive tumor cells), 1 (< 10% positive tumor cells), 2 (10%–50% positive tumor cells), and 3 (> 50% positive tumor cells). The intensity of protein expression was evaluated at 4 levels: negative = 0, weak = 1, moderate = 2, and strong = 3. The final immunoreactivity score was calculated by multiplying the staining intensity and density scores, thus resulting in scores of 0, 1, 2, 3, 4, 6, or 9. A final score ≥ 4 was considered to indicate high expression, and a score < 4 was considered to indicate low expression.

### Plasmid construction, transfection, and cell sorting

Stable short hairpin RNA (shRNA) knockdown of *L1CAM* (with shL1CAM) in EC1 cells was performed with the pGV248-hu6-GFP-Puro vector plasmid (Gene Pharma, Shanghai, China) according to the manufacturer’s instructions. EC1 cells transfected with empty vector (shNC) served as a negative control. *L1CAM* was stably overexpressed (OE-L1CAM) in KYSE450 cells with the pCDH-EF1-MCS-T2A-GFP vector (SBI, USA) encoding the *L1CAM* cDNA sequence. KYSE450 cells transfected with the empty vector were used as controls (Scramble). The cells were then transfected with the plasmid with Lipofectamine 3000 (Invitrogen, Waltham, MA, USA). Viral particles were harvested 48 h after transfection, and cells were infected with virus together with polybrene (10 mg/mL, Sigma, St. Louis, MO, USA). The expression of *L1CAM* was confirmed by qRT-PCR and Western blot. The transfected cells were sorted with a MoFlo XDP instrument (Beckman, CA, USA) according to the expression of green fluorescent protein, and the purity was found to be above 95%.

### Western blot

Detailed experiments were performed as previously described^6^. Total cell protein was extracted with RIPA lysis buffer (Beyotime, Shanghai, China). Equal amounts of protein (50 μg) were loaded on 10% SDS–PAGE gels. After electrophoresis, the proteins were blotted onto a polyvinylidene fluoride membrane. The membrane was blocked in 5% nonfat milk and incubated with primary antibodies overnight at 4 °C, then with secondary antibody (Cell Signaling Technology, Danvers, MA, USA) for 2 h at 37 °C. Primary antibodies against the following proteins were used: L1CAM (Santa Cruz Biotechnology, CA, USA), CCL22 (R&D Systems, USA), β-actin, pAkt, Akt, pNF-κB, NF-κB, pSmad2, Smad2, pSmad3, and Smad3 (Cell Signaling Technology).

### Cell proliferation assay

A CCK-8 kit (Dojindo Corp., Kumamoto, Japan) was used according to the manufacturer’s instructions. Cells were seeded in 96-well plates at a density of 1 × 10^3^ cells per well. After every 24 h for 4 days, 10 μL of CCK-8 reagent was added per well and incubated at 37 °C for 1 h, after which the absorbance was measured at 450 nm (MULTISKANMK3, Thermo Fisher Scientific Inc., Waltham, MA, USA).

### Cell cycle analysis

Cells were harvested and washed with chilled PBS, then fixed with 70% alcohol overnight at 4 °C. The cells were then washed with PBS, treated with RNase for 60 min, and stained with propidium iodide (Sigma, St. Louis, MO, USA) for 30 min at 37 °C. Cell cycle analysis was performed *via* flow cytometry and analyzed in ModFit LT5.0 software (Coulter).

### Apoptosis assay

Cells were harvested and washed with chilled PBS, collected, and suspended in Annexin V binding buffer (BioLegend, USA). Next, the cells were incubated with 1 μL Annexin V Alexa Fluor 647 (BioLegend, USA) for 15 min at 4 °C in the dark, and this was followed by the addition of propidium iodide. Samples were immediately analyzed with flow cytometry.

### Isolation of CD4^+^CD25^+^ Treg cells

Isolation of CD4^+^CD25^+^ T cells from the peripheral blood of patients with ESCC was performed with CD4 and CD25 magnetic beads according to the manufacturer’s instructions (Miltenyi Biotech, Germany). Next, the isolated cells were sorted with CD4^+^CD25^+^ with a MoFlo XDP instrument (Beckman, CA, USA). The sorted CD4^+^CD25^+^ T cells were prepared for CD127^-^ cell sorting and used for cell function experiments. Cell populations were found to be more than 90% pure by flow cytometry analysis.

### Chemotaxis assay

The chemotaxis assays were performed as previously described^14^. The chemotactic migration of CD4^+^CD25^+^ CD127^-^ Tregs was evaluated in 24-well Transwell plates with 5 μm pore size polycarbonate filters (Corning Inc, Coring, NY, USA). A total of 1 **×** 10^5^ freshly isolated Tregs from patient peripheral blood were seeded into the upper chamber. Tumor cells, anti-CCL22 neutralizing antibodies (500 ng/mL; R&D, MAB336) or CCL22 recombinant protein (100 ng/mL, BioLegend, 584902) was added to the lower chamber. Tregs that had migrated into the lower chamber were counted after 24 h.

### *In vitro* migration and invasion assays

Migration and invasion assays were assessed as described previously^15^. Tumor cells (1 **×** 10^5^) in 200 μL serum-free medium were added into the upper chamber (8.0 μm pore size, 24-well insert), and Matrigel (BD Biosciences, San Jose, CA, USA) was plated in the wells for the invasion assay. Medium containing fetal bovine serum (600 μL) was then added to the lower chamber, and the cells were incubated under standard culture conditions either for 24 h (migration assay) or for 48 h (invasion assay). The cells remaining on the upper surface of the membrane were then removed, and the cells that had migrated through the membrane were stained with 0.2% crystal violet, imaged, and counted under a microscope (Olympus, Tokyo, Japan).

### Enzyme-linked immunosorbent assay (ELISA)

Cell culture supernatants were obtained from the plates of treated cells, and ELISAs were performed with Human CCL22 and Human TGF-β1 ELISA kits (BioLegend, USA), according to the manufacturer’s instructions. Absorbance was measured at 450 nm with a microplate reader (MULTISKANMK3, Thermo Fisher Scientific Inc., Waltham, MA, USA).

### Multiplex bead-based assay

Human chemokine and cytokine detection was performed with a LEGENDplexTM Multi-Analyte Flow Assay Kit (BioLegend, USA, #740001). Supernatants were collected from the same quantity of the indicated cells and were centrifuged before analysis. Results were normalized to standards provided in the BioLegend LEGENDplexTM kit. Detailed experiments were performed according to the standard protocol.

### Tumor formation in BALB/c nude mice

For generation of a subcutaneous xenograft mouse model, we randomly divided 20 female BALB/c nude mice (Vital River Laboratory Animal Technology Co. Ltd, Beijing, China) 5 weeks of age into 4 groups (five mice per group). All animal experiments were performed in accordance with the Guide for the Care and Use of Laboratory Animals of Zhengzhou University. All the xenograft mouse model experiments were conducted in the Henan Key Laboratory for Pharmacology of Liver Diseases, and the animals’ certificate were approved by the ethics committee of Henan Key Laboratory for Pharmacology of Liver Diseases (Approval No. 2019-41). Each group received hypodermic injections of shNC-EC1, shL1CAM-EC1, scramble-KYSE450, or OE-L1CAM KYSE450 cells on the right flank (5 × 10^6^ tumor cells in 100 μL of PBS). The tumor dimensions were measured as length (L) and width (W) with calipers every 3 days, and the volumes were calculated with the formula (L × W^2^)/2. Mice were euthanized by cervical dislocation after being anesthetized with 10% chloral hydrate on day 21 or 27, and the tumors were excised and snap-frozen for RNA extraction. IHC of the tumor tissues was performed as described above.

### *In vivo* migration

Detailed experiments were performed as previously described^16^. For the recruitment of Tregs in the *in vivo* assay, shNC-EC1, shL1CAM-EC1, scramble-KYSE450, and L1CAM-OE KYSE450 cells in 100 μL PBS were inoculated subcutaneously in the right flank, respectively. On days 23 and 26 after tumor inoculation, the nude mice were randomly selected for treatment *via* intraperitoneal injection with the IgG control (IgG2b: R&D Systems) or CCL22 neutralizing antibody (500 ng/mL; R&D, MAB336). On day 25, 100 μL human CD4^+^CD25^+^ Tregs were injected into the tail veins. On day 27, all mice were euthanized to obtain tumor tissues for RNA extraction, IHC, and assessment of the percentage of CD4^+^FOXP3^+^ and FOXP3^+^CCR4^+^ Tregs recruited to the tumor site with flow cytometry.

### The Cancer Genome Atlas (TCGA) database analysis

The mRNA expression levels of *L1CAM*, *CCL22*, and *FOXP3* were obtained from TCGA (https://cancergenome.nih.gov/), *n* = 94. All patients with ESCC were treatment naive. The patients were divided into 2 groups on the basis of the median value of the *L1CAM* mRNA expression level. The R package “limma” was used to calculate the differentially expressed genes (DEGs) with threshold values of *P* < 0.05 and logFc >1.5.

### Statistical analysis

Data analysis was performed in GraphPad Prism 7 (GraphPad Software, La Jolla, CA, USA) and SPSS 21 software (SPSS, Chicago, IL, USA). Data are expressed as the mean ± standard deviation of at least 3 independent experiments. Student’s *t*-test was performed to analyze the differences between groups with normally distributed continuous variables. Pearson’s coefficient correlation or linear regression analysis was used to analyze the relationships between the expression levels of specific genes. OS was defined as the date from surgery to the date of death or the last follow-up. Progression free survival (PFS) was calculated as the date from the surgery to the date of disease progression or last follow-up. OS and PFS were evaluated with the Kaplan-Meier method. In all cases, *P* values < 0.05 were considered statistically significant.

## Results

### L1CAM expression is significantly elevated in ESCC and correlates with poorer prognosis

**Figure 1:**
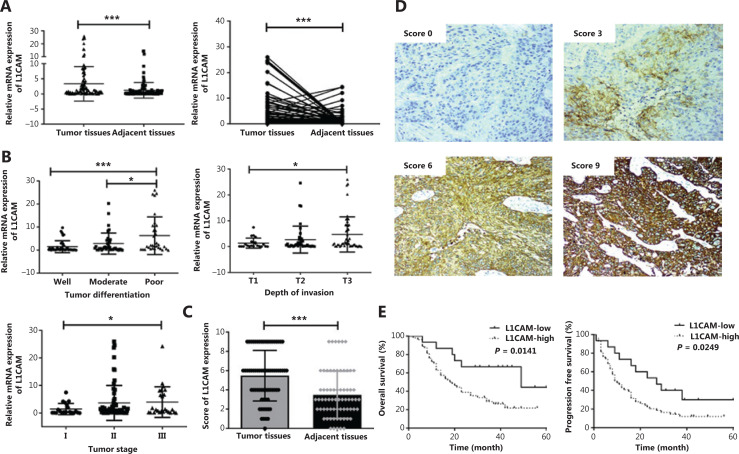
Expression of L1CAM is elevated in ESCC tissues. (A) qRT-PCR analysis of L1CAM mRNA expression in paired fresh tissues from 106 patients with ESCC. GAPDH was used to normalize the data, which were analyzed with the 2^-ΔΔCt^ method. (B) Samples were divided according to their tumor differentiation, depth of invasion, and tumor stage, and the mRNA expression of L1CAM in ESCC tissues was analyzed. (C) IHC score analysis of L1CAM protein expression in 69 ESCC and paired adjacent normal tissues (***P < 0.001). (D) Representative images showing different intensities of L1CAM staining (200×). (E) Kaplan-Meier survival analysis of OS and PFS, on the basis of high (*n* = 54) and low (*n* = 15) L1CAM protein expression *via* IHC. *P < 0.05, ***P < 0.001.

To study the clinical importance of L1CAM expression, we examined the *L1CAM* mRNA levels in 106 fresh tissue samples with paired adjacent normal tissues from patients with ESCC by using qRT-PCR. As shown in **Figure 1A**, the expression levels of *L1CAM* mRNA were significantly higher in ESCC tissues than in adjacent normal tissues (*P* = 0.0005). The expression of *L1CAM* mRNA was closely correlated with the tumor differentiation, depth of invasion, and tumor stage (*P* < 0.05 or *P* < 0.001, **Figure 1B**), but was not significantly correlated with age, sex, lymph node metastasis, or other clinical parameters (data not shown).

To investigate whether L1CAM expression might be significantly associated with the long-term prognosis of patients, we first examined L1CAM expression in 69 ESCC and adjacent normal tissues at the protein level by using IHC. The results confirmed that L1CAM protein was highly expressed in ESCC tissues (**Figure 1C**); selected representative IHC images are shown in **Figure 1D**. On the basis of the level of L1CAM protein expressed in their ESCC tissues, 69 patients were then divided into L1CAM high and low expression groups, and statistical analysis of clinical characteristics was performed. L1CAM protein expression in tumor tissues was associated with T classification, differentiation, tumor/node/metastasis (TNM) stage, and survival status (**Table 1**). Patients with higher L1CAM protein expression had poorer OS and PFS than patients exhibiting lower L1CAM expression (**Figure 1E**). Both univariate and multivariate survival analyses indicated that TNM stage and L1CAM expression were independent prognostic factors for overall survival of patients with ESCC (*P* < 0.001 and *P* = 0.045, **Table 2**). The above results indicated that L1CAM is overexpressed in ESCC tissues and is closely associated with patient prognosis.

**Table 1:** Clinicopathological features and L1CAM expression in patients with esophageal squamous cell carcinoma

Characteristics	Total No.	L1CAM expression		*χ*^2^	*P*
		High	Low
Age (years)				1.193	0.275
≥ 60	45	37	8		
< 60	24	17	7		
Gender				0.417	0.518
Male	41	31	10		
Female	28	23	5		
T classification				4.813	0.040
T1–2	38	26	12		
T3	31	28	3		
Differentiation status				4.299	0.045
Well-moderate	39	27	12		
Poor	30	27	3		
TNM stage				7.379	0.007
I	18	10	8		
II–IV	51	44	7		
Lymph node invasion				0.757	0.384
Absent	30	22	8		
Present	39	32	7		
Survival status				6.133	0.013
Alive	23	14	9		
Dead	46	40	6		

**Table 2:** Univariate and multivariate analysis of various potential prognostic factors in patients with esophageal squamous cell carcinoma

Characteristics	Univariate analysis			Multivariate analysis	
	Median OS, mo	HR (95% CI)	*P*	HR (95% CI)	*P*
Gender					
Male	23	0.898 (0.491–1.628)	0.713		
Female	20.5				
Age (years)					
≥ 60	20	1.339 (0.724–2.460)	0.346		
< 60	34				
TNM stage					
I–II	49	0.153 (0.064–0.243)	< 0.0001	0.168 (0.072–0.363)	< 0.001
III–IV	12				
T classification					
T1–2	25	0.873 (0.478–1.575)	0.640		
T3	19				
Differentiation status					
Well-moderate	25	0.567 (0.295–0.995)	0.048	0.777 (0.441–1.446)	0.435
Poor	16				
Lymph node invasion					
Absent	49	0.319 (0.176–0.593)	< 0.001	0.698 (0.337–1.446)	0.333
Present	17				
L1CAM expression					
Low	49	0.361 (0.262–0.920)	0.026	0.371 (0.140–0.978)	0.045
High	18				

### Knockdown or overexpression of L1CAM influences the malignant behavior of ESCC cells

**Figure 2:**
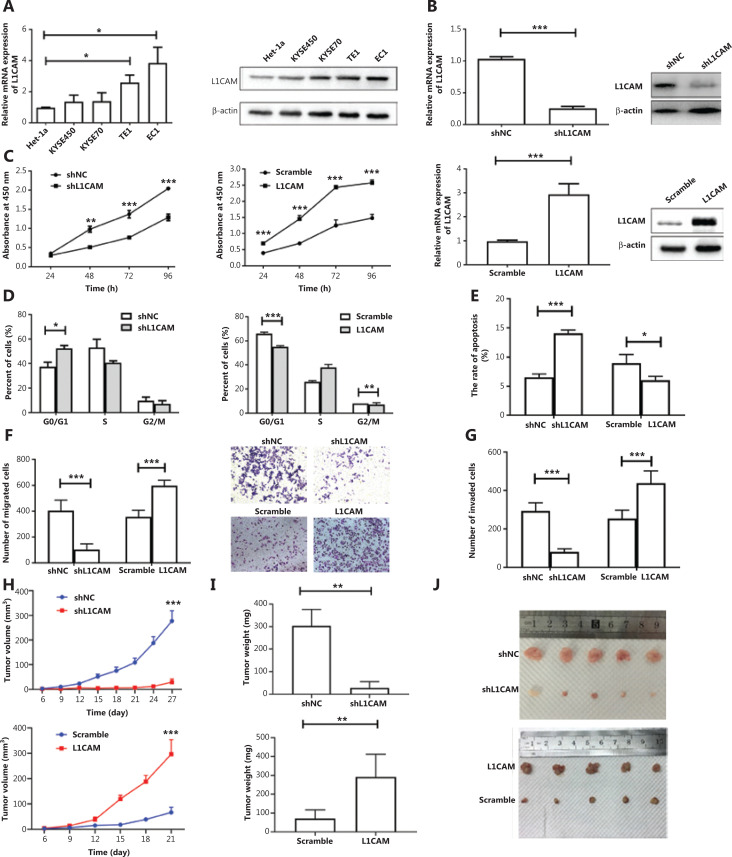
L1CAM promotes ESCC tumor malignancy. (A) The mRNA and protein levels of L1CAM, examined with qRT-PCR and Western blot, in one immortalized esophageal cell line (Het-1α) and 4 human ESCC cell lines (KYSE450, KYSE70, TE1, and EC1). (B) L1CAM expression was detected with qRT-PCR and Western blot in shNC, shL1CAM-EC1 cells, scramble, and OE-L1CAM-KYSE450 cells. The effects of L1CAM on the proliferation of EC1 and KYSE450 cell lines were analyzed with CCK-8 (C), cell cycle (D), and apoptosis (E) assays. Summary bar chart (left) and representative images (right) of cell migration and invasion abilities in shNC, shL1CAM-EC1 cells, scramble, and OE-L1CAM-KYSE450 cells detected with Transwell (F) and Matrigel invasion (G) assays. Cells present in the substrate were stained with crystal violet and counted under a microscope (200×). The volumes (H) and weights (I) of the tumors grown in the xenograft mouse model are presented as the mean ± standard deviation (*n* = 5). (J) Photographs of the dissected tumors from different groups *P < 0.05, **P < 0.01, ***P < 0.001.

To explore the biological function of L1CAM in esophageal carcinoma, we used qRT-PCR to assess the* L1CAM* expression in 4 ESCC cell lines (EC1, KYSE70, TE1, and KYSE450) and an immortalized esophageal epithelial cell line, Het-1α. *L1CAM* mRNA expression was significantly higher in TE1 and EC1 cells. According to the results of Western blot (**Figure 2A**), we selected cell lines with higher and lower L1CAM expression, EC1 and KYSE450, respectively, for further research.

To investigate the effect of L1CAM on the development and progression of ESCC, we stably transfected EC1 cells with shNC or L1CAM-specific shRNA (shL1CAM), and KYSE450 cells with an L1CAM overexpression (OE-L1CAM) or scramble control vector. Transfection efficiency was measured *via* qRT-PCR and Western blot (**Figure 2B**). The CCK-8 assay was used to examine the effect of L1CAM on the proliferation of ESCC cells. Notably, L1CAM knockdown dramatically decreased the growth rate of EC1 cells, whereas its overexpression increased the growth rate of KYSE450 cells, as compared with that of the negative control (**Figure 2C**). The effects of L1CAM on the cell cycle and apoptosis of ESCC cells were measured with flow cytometry. After knockdown of L1CAM, the proportion of KYSE450 cells in G0/G1 phase markedly increased, and a corresponding decrease in the S phase population was observed, whereas L1CAM overexpression decreased the proportion of EC1 cells in G0/G1 and G2/M phases (**Figure 2D**). Annexin V/PI staining demonstrated that the rate of apoptosis in the EC1 cells was significantly lower than that in the empty lentiviral vector control cells (**Figure 2E**). Moreover, the results from Transwell assays showed that silencing of L1CAM significantly inhibited the migration and invasion of EC1 cells (**Figure 2F and 2G**). However, the ectopic overexpression of L1CAM markedly increased the proliferation, apoptosis, migration, and invasion of KYSE450 cells (**Figure 2C, 2D, 2E, 2F, and 2G**). To further confirm the role of L1CAM *in vivo*, we established a BALB/c nude mouse xenograft model by using shL1CAM and OE-L1CAM cells. First, we evaluated *in vivo* tumor growth by measuring the tumor size and weight. Notably, mice injected with shL1CAM EC1 cells developed tumors that were smaller in both size and weight than the tumors in mice injected with control cells, whereas mice injected with OE-L1CAM KYSE450 cells exhibited tumors of greater size and weight (**Figure 2H, 2I, and 2J**). These data provide further evidence that L1CAM plays an oncogenic role in ESCC *in vivo*.

### L1CAM promotes the expression of CCL22

**Figure 3:**
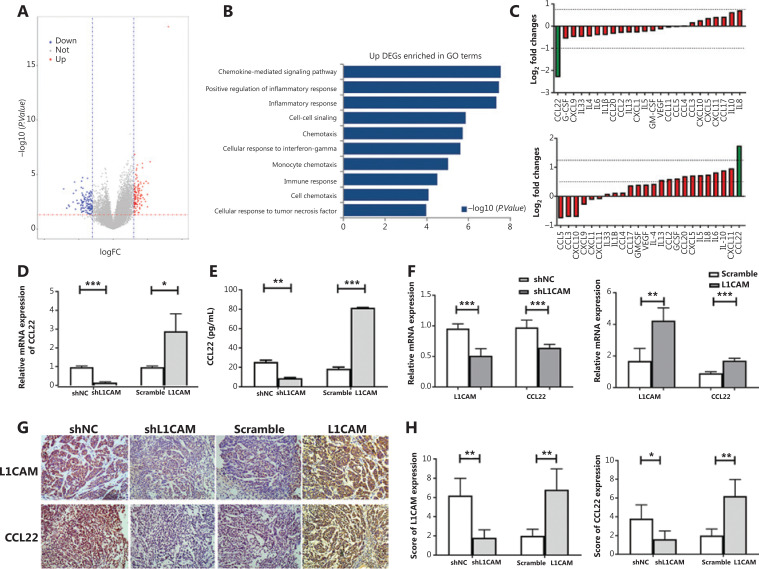
L1CAM promotes the recruitment of Tregs by upregulating CCL22. (A) Volcano plot of differentially expressed genes (DEGs), on the basis of the median value of the L1CAM mRNA expression level. (B) The upregulated DEGs were used to perform GO enrichment analysis. (C) The secretion of cytokines and chemokines in the supernatants of shNC and shL1CAM-EC1 cells (up) or scramble and OE-L1CAM-KYSE450 cells (down) cultured for 48 h was assessed with a human multiplex bead-based kit. The expression of CCL22 was detected by qRT-PCR (D) and ELISA (E) between shNC and shL1CAM-EC1 cells, or scramble and OE-L1CAM-KYSE450 cells. (F) Expression of L1CAM and CCL22 mRNAs in the different groups tumor samples derived from **Figure 2H**. (G) IHC of tumor tissues in different groups derived from **Figure 2H**. Representative images are shown (200×). (H) The IHC scores of L1CAM and CCL22 in different groups. *P < 0.05, **P < 0.01, ***P < 0.001.

Emerging evidence indicates that the TME plays a crucial role in the development of solid cancers^8,17^. To assess the interaction between L1CAM expression and the TME, we divided the patients with ESCC selected from TCGA database into *L1CAM* high and *L1CAM* low groups, on the basis of the median value of *L1CAM* mRNA expression, and identified the DEGs. Then we used the DEGs to perform GO enrichment analysis. The results suggested that *L1CAM* mainly regulates immunity and the chemokine pathways (**Figure 3A and 3B**). To further identify the factors mediating communication between cancer and immune cells in this context, we performed cytokine array analysis with a human multiplex bead-based kit and found that CCL22 was significantly downregulated in shL1CAM cells (**Figure 3C**, upper panel), whereas it was significantly upregulated in L1CAM overexpressing cells at the protein level (**Figure 3C**, lower panel). These results were confirmed by qRT-PCR and ELISA (**Figure 3D and 3E**). Subsequently, to further confirm these findings, we examined CCL22 expression in the indicated subcutaneous tumor samples derived from **Figure 2J** by using qRT-PCR and IHC. The expression of CCL22 was significantly lower in shL1CAM EC1 cells than shNC cells, whereas it was higher in OE-L1CAM KYSE450 cells (**Figure 3F, 3G and 3H**). The expression of CCL22 paralleled that of L1CAM. The* in vivo* results were also consistent with the *in vitro* results. Together, these results suggest that L1CAM may promote the expression of CCL22.

### L1CAM facilitates recruitment of Tregs through CCL22

**Figure 4:**
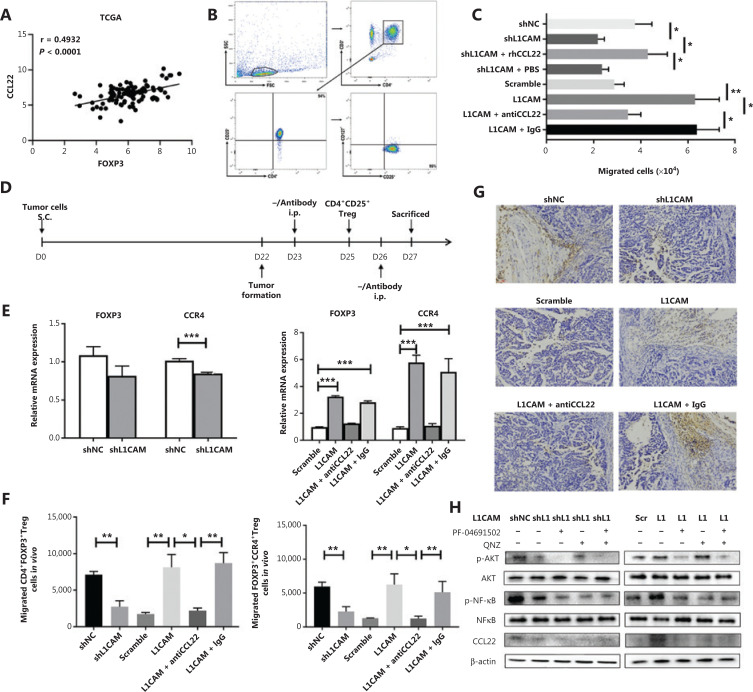
L1CAM promotes the recruitment of Tregs through CCL22 *in vivo*. (A) The correlation between FOXP3 and CCL22 in the ESCC TCGA database, *n* = 94. (B) The purity of CD4^+^CD25^+^CD127^-^ Tregs sorted from the peripheral blood of patients with ESCC, according to FACS. (C) The numbers of migrating Tregs obtained from ESCC patient blood samples was calculated for the shNC, shL1CAM, shL1CAM + recombinant protein CCL22 (100 ng/mL), shL1CAM + PBS, scramble, OE-L1CAM, OE-L1CAM + CCL22 antibody (500 ng/mL), and OE-L1CAM + IgG groups. (D) Flow chart of the *in vivo* experiments. (E) mRNA expression of FOXP3 and CCR4 in the indicated groups. (F) Percentage of CD4^+^FOXP3^+^ Tregs and FOXP3^+^CCR4^+^ Tregs recruited to tumor sites, as analyzed by flow cytometry. (G) Expression of FOXP3 in tumor tissues of different groups, determined by IHC (200×). (H) The expression of p-Akt, Akt, p-NF-κB, NF-κB, and CCL22 was detected by Western blot between shL1CAM and OE-L1CAM cells treated with or without p-Akt inhibitor (RF-04691502) and p-NF-κB inhibitor (QNZ). β-actin expression was used as a loading control. *P < 0.05, **P < 0.01, ***P < 0.001.

CCL22 has been implicated in the recruitment of CCR4^+^ Tregs from peripheral blood to tumor tissues^18,19^. We found a positive correlation between FOXP3 and CCL22 in TCGA database of ESCC, *n* = 94 (**Figure 4A**). Therefore, we determined whether high L1CAM expression might result in recruitment of Tregs. First, we obtained CD4^+^CD25^+^CD127^-^ Tregs from the peripheral blood of patients with ESCC and assessed cell populations with more than 90% purity by using FACS (**Figure 4B**). The results of the classical Transwell migration assay showed that the number of Tregs chemoattracted to the cell supernatant was lower in shL1CAM than shNC cells, but this difference was negated by the addition of CCL22 recombinant protein. Conversely, more Tregs were attracted to the cell supernatants of L1CAM-overexpressed cells compared with scramble control cells, but this difference was abrogated by CCL22-neutralizing antibody (**Figure 4C**). To further investigate whether CCL22 mediates the role of L1CAM in the recruitment of Tregs *in vivo*, we designed the study in **Figure 4D**. We found that the expression of FOXP3 and CCR4, and the percentage of CD4^+^FOXP3^+^ and FOXP3^+^CCR4^+^ Tregs chemoattracted to the tumor sites were significantly downregulated in the shL1CAM group, but upregulated in the L1CAM overexpression group, as compared with the control. Furthermore, these effects were attenuated by CCL22-neutralizing antibody *in vivo* (**Figure 4E and 4F**). IHC also showed the same results (**Figure 4G**). Together, these results indicate that L1CAM recruits Tregs to tumor sites, thereby promoting tumor progression by regulating CCL22 secretion *in vitro* and *in vivo.*

To further explore the mechanism by which L1CAM upregulates the expression of CCL22, we performed Western blot assays to examine the regulation of the signaling pathway. The phosphorylation of Akt/NF-κB (p-Akt/NF-κB) was activated in L1CAM overexpressing cells compared with scramble control cells, and inhibition of p-Akt/NF-κB was observed in shL1CAM cells (**Figure 4H**). Thus, we postulated that L1CAM increases the expression of CCL22 in a p-Akt/NF-κB-dependent manner. Therefore, we used inhibitors of p-Akt and p-NF-κB, PF-04691502 and QNZ, to suppress the activation of p-Akt/NF-κB in tumor cells. Both the p-Akt and p-NF-κB inhibitors abolished CCL22 expression in OE-L1CAM and shL1CAM cells (**Figure 4H**). These data suggest that L1CAM upregulates CCL22 by activating the PI3K/Akt/NF-κB signaling pathway.

### Tregs secrete TGF-β, which in turn induces L1CAM expression by activating Smad2/Smad3

**Figure 5:**
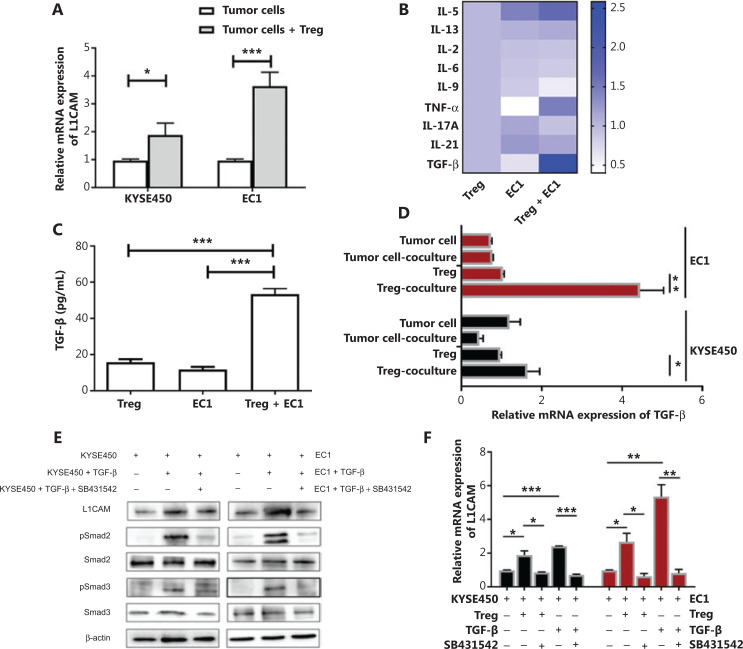
Tregs secrete TGF-β, which in turn induces L1CAM expression by activating Smad2/Smad3. (A) L1CAM expression in KYSE450 and EC1 cells with or without co-culturing with Tregs (1:1), as determined by qRT-PCR. (B) A multiple-cytokine kit was used to detect cytokines in the supernatants from the EC1/Treg co-culture system and from the EC1 and Tregs cultured alone. (C) The TGF-β protein level was validated using ELISA. (D) TGF-β expression in the tumor cells and Tregs obtained from the co-culture system, and in the tumor cells and Tregs cultured alone. (E) EC1 and KYSE450 cells pretreated with or without TGF-β recombinant protein (20 ng/mL) or TGF-β inhibitor (SB431542) (10 μM). The expression of L1CAM, p-Smad2, Smad2, p-Smad3, and Smad3 was examined with Western blot analysis. β-actin expression was used as a loading control. (F) L1CAM expression was detected in tumor cells with or without Tregs, rhTGF-β, or SB431542. *P < 0.05, **P < 0.01, ***P < 0.001.

To investigate the interaction between Tregs and tumor cells, we co-cultured Tregs with KYSE450 or EC1 cells. Tregs increased L1CAM expression in both KYSE450 and EC1 cells (**Figure 5A**). To understand how Tregs induce L1CAM expression in tumor cells, we analyzed the supernatants from the Tregs or EC1 cells cultured alone, or from the EC1 and Treg co-culture system. We performed cytokine array analysis and found that the most markedly elevated cytokine in the co-culture system was TGF-β (**Figure 5B**). A similar result was observed at the TGF-β protein level by using ELISA (**Figure 5C**). To identify the source of TGF-β, we performed qRT-PCR to determine the TGF-β expression in tumor cells and Tregs obtained separately from the co-culture system, and in the non-co-cultured tumor and Treg cells. The mRNA expression of TGF-β was higher in Tregs obtained from the co-culture system with EC1 and KYSE450 than in the other groups (**Figure 5D**). To further address whether TGF-β enhanced L1CAM expression, we treated KYSE450 and EC1 with TGF-β recombinant protein. Both the mRNA and protein levels of L1CAM increased after TGF-β treatment, whereas this effect was abrogated by the TGF-β inhibitor SB431542 (**Figure 5E**). Next, we examined the mechanism through which TGF-β increases the expression of L1CAM. After TGF-β binding, the receptor complex phosphorylates the transcription factors Smad2 and Smad3, which then bind Smad4 and accumulate in the nucleus, where they regulate target gene transcription^20^. In line with findings from previous reports, we observed that TGF-β activated Smad2 and Smad3 in KYSE450 and EC1 cells, and this effect was abolished by TGF-β inhibitor treatment (**Figure 5E**). To further address whether TGF-β might be implicated in Treg-enhanced L1CAM expression, we included a TGF-β inhibitor in the Treg and tumor cell co-culture assay. The increased expression of L1CAM induced by Tregs was attenuated by the TGF-β inhibitor (**Figure 5F**). These results suggest that Tregs contribute to L1CAM expression through the TGF-β-Smad2/Smad3 signaling pathway.

### High expression of CCL22 predicts poor survival in patients with ESCC

**Figure 6:**
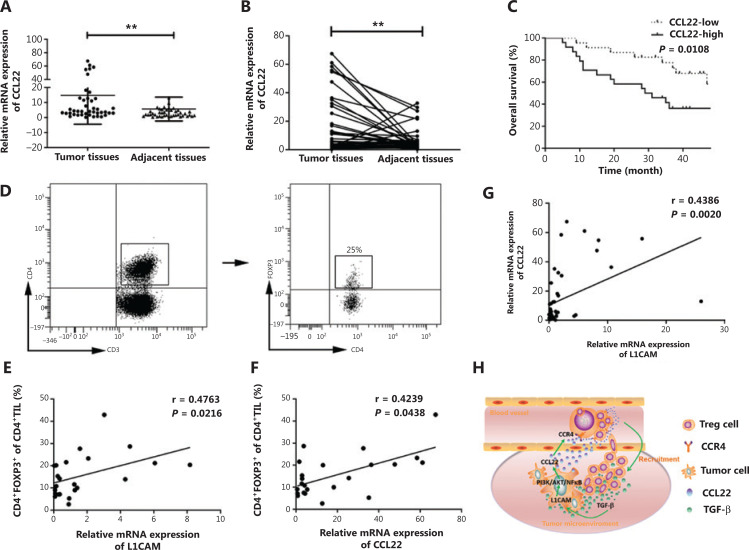
High expression of CCL22 predicts poor survival in patients with ESCC. (A–B) qRT-PCR analysis of CCL22 expression in paired fresh tissues from 47 patients with ESCC. GAPDH was used to normalize the data, which were analyzed with the 2^-ΔΔCt^ method. (C) Kaplan–Meier survival analysis of overall survival on the basis of high (*n* = 24) and low (*n* = 23) CCL22 expression by qRT-PCR. (D) The percentage of CD4^+^FOXP3^+^ Tregs in tumor tissues obtained from patients with ESCC. Correlations between CD4^+^FOXP3^+^ Tregs and L1CAM (E), CD4^+^FOXP3^+^ Tregs, and CCL22 (F), L1CAM, and CCL22 in the tumor tissues of patients with ESCC (G) were evaluated with linear regression analysis. (H) Schematic diagram of the proposed molecular mechanism: L1CAM promotes the recruitment of Tregs into the tumor site through PI3K/Akt/NF-κB-CCL22-CCR4. Tregs produce TGF-β, which further induces L1CAM upregulation in ESCC tumor cells. **P < 0.01.

Finally, we investigated the clinical relevance of CCL22 in patients with ESCC. We found that CCL22 expression was higher in ESCC tumor tissues than in adjacent normal tissues (**Figure 6A and 6B**). Kaplan-Meier curve analysis was performed to analyze the correlation between CCL22 and the survival of patients of ESCC. When we divided patients into groups with low and high median expression values of CCL22, we found that high CCL22 expression was associated with poorer patient survival (**Figure 6C**). Additionally, the percentage of CD4^+^FOXP3^+^ Tregs in tumor tissues (**Figure 6D**) exhibited a significant positive association with L1CAM and CCL22 in patients with ESCC (**Figure 6E and 6F**). There was also a significant positive association between L1CAM and CCL22 (**Figure 6G**).

## Discussion

L1CAM was originally identified as a crucial regulator in the development of the nervous system. Subsequent studies revealed that L1CAM is elevated in different types of human cancers, including pancreatic neuroendocrine tumors, glioblastoma, and colorectal cancer^21^. L1CAM has also been identified as a prognostic marker, a tool for diagnostics, and most importantly as a potential therapeutic target in cancer treatment^22–24^. In this study, we found that the expression of L1CAM was higher in tumor tissues than in adjacent tissues and was correlated with tumor differentiation and tumor stage in ESCC. We also found that L1CAM promoted the oncogenesis of ESCC *in vitro* and* in vivo.* Tang et al.^25^ have also reported that L1CAM promotes tumor growth and metastasis in ESCC, a finding in line with the results from our study.

Recent emerging evidence indicates that the TME, particularly immune cells, plays a role in the progression of tumors^26^. Studies investigating L1CAM have focused on its tumorigenicity, although little attention has been paid to potential interactions with the TME. Therefore, we investigated whether L1CAM regulates TME in our study. Various chemokines and cytokines in the TME have been reported to play crucial roles in tumor progression, and indeed, when we used the DEGs to perform GO enrichment analysis, we found that L1CAM was associated with immunity and the chemokine pathway. Therefore, we performed a multi-cytokine assay to detect the expression of chemokines and found that CCL22 levels were significantly altered in shL1CAM and L1CAM overexpressing cells. Further investigation confirmed a significant positive correlation between L1CAM and CCL22, and revealed that L1CAM regulates CCL22 expression by activating the PI3K/Akt and NF-κB signaling pathways.

Evidence from recent studies suggests that CCL22 plays a role in recruiting Tregs to tumor sites by binding the CCR4 receptor, which further facilitates tumor progression^27^. The accumulation of Tregs in the TME inhibits the immune response against tumors and is regarded as an important mechanism through which tumors escape immunosurveillance^19^. Increased infiltration of CCR4^+^ Tregs is associated with poor prognosis in different types of cancers, such as pancreatic ductal adenocarcinoma^28^, prostate cancer^29^, and colorectal cancer^17^. The depletion of Tregs has been shown to facilitate the induction of antitumor responses in murine models^30^. Depletion of CCR4^+^FOXP3^+^CD4^+^ Tregs by KW-0761, a humanized anti-CCR4 monoclonal antibody, was investigated in patients with solid tumors in a phase Ia clinical trial^31,32^. Blocking the CCL22-mediated recruitment of CCR4^+^ Tregs may provide a potential strategy to control tumor growth. In the current study, we found that L1CAM regulates the recruitment of Tregs by affecting CCL22 secretion. The NF-κB signaling pathway activated by L1CAM in this process is also important, because it is known to promote cell proliferation *in vitro* and metastasis *in vivo*^33,34^*.* We observed that L1CAM activated the PI3K/Akt and NF-κB pathways, thereby inducing CCL22 expression. On the basis of these results, L1CAM may serve as a potential therapeutic target for patients with ESCC, because it not only inhibits tumor cell growth but also diminishes Treg infiltration.

Tregs promote immune escape mainly by secreting various soluble factors such as IL-10 and TGF-β. TGF-β is an immunosuppressive cytokine that plays an important role in tumor initiation and progression. TGF-β ligands signal through type I and type II TGF-β receptors (TβRI and TβRII, respectively). After binding of TGF-β to TβRII, TβRI is recruited, trans-phosphorylated, and activated; it in turn phosphorylates the downstream mediators Smad2 and Smad3^35^. Several reports have focused on the relationship between TGF-β and L1CAM. After stimulation by TGF-β, the transcription factor Slug binds the L1CAM promoter and increases its expression in colonic intestinal epithelial cells, thus enhancing cell motility and apoptosis resistance^36^. The expression of L1CAM is increased by TGF-β treatment in endometrial and pancreatic cancer cells in a SLUG-dependent fashion^36,37^. In our study, we also found that TGF-β derived from Tregs promotes L1CAM expression in ESCC cell lines by activating the Smad2/Smad3 signaling pathway.

## Conclusions

In conclusion, L1CAM promotes tumorigenesis and induces CCL22 secretion in ESCC tumor cells *via* PI3K/Akt and NF-κB. CCL22 further promotes the recruitment of Tregs into tumor sites. Tregs produce high levels of TGF-β, which in turn further upregulates L1CAM expression in tumor cells and promotes tumor progression (**Figure 6H**). These findings suggest a crucial role of L1CAM in TME and provide a novel therapeutic strategy for patients with ESCC.
